# The current state of intermittent intraoperative neural monitoring for prevention of recurrent laryngeal nerve injury during thyroidectomy: a PRISMA-compliant systematic review of overlapping meta-analyses

**DOI:** 10.1007/s00423-017-1580-y

**Published:** 2017-04-04

**Authors:** Brandon Michael Henry, Matthew J. Graves, Jens Vikse, Beatrice Sanna, Przemysław A. Pękala, Jerzy A. Walocha, Marcin Barczyński, Krzysztof A. Tomaszewski

**Affiliations:** 1 International Evidence-Based Anatomy Working Group, 12 Kopernika St, 31–034 Krakow, Poland; 2 Department of Anatomy, Jagiellonian University Medical College, 12 Kopernika St, 31–034 Krakow, Poland; 3 Division of Medicine, Stavanger University Hospital, Gerd-Ragna Bloch Thorsens gate 8, 4011 Stavanger, Norway; 4 Faculty of Medicine and Surgery, University of Cagliari, S.S. 554, Bivio Sestu, 09042 Monserrato, CA, Sardinia Italy; 5 Department of Endocrine Surgery, Third Chair of General Surgery, Jagiellonian University Medical College, 35-37 Pradnicka St, 31–202 Krakow, Poland

**Keywords:** Intermittent intraoperative nerve monitoring, Recurrent laryngeal nerve, Thyroidectomy, Vocal fold paresis, Iatrogenic injury

## Abstract

**Purpose:**

Recurrent laryngeal nerve (RLN) injury is one of the most common and detrimental complications following thyroidectomy. Intermittent intraoperative nerve monitoring (I-IONM) has been proposed to reduce prevalence of RLN injury following thyroidectomy and has gained increasing acceptance in recent years.

**Methods:**

A comprehensive database search was performed, and data from eligible meta-analyses meeting the inclusion criteria were extracted. Transient, permanent, and overall RLN injuries were the primary outcome measures. Quality assessment via AMSTAR, heterogeneity appraisal, and selection of best evidence was performed via a Jadad algorithm.

**Results:**

Eight meta-analyses met the inclusion criteria. Meta-analyses included between 6 and 23 original studies each. Via utilization of the Jadad algorithm, the selection of best evidence resulted in choosing of Pisanu et al. (Surg Res 188:152–161, 2014). Five out of eight meta-analyses demonstrated non-significant (*p* > 0.05) RLN injury reduction with the use of I-IONM versus nerve visualization alone.

**Conclusions:**

To date, I-IONM has not achieved a significant level of RLN injury reduction as shown by the meta-analysis conducted by Pisanu et al. (Surg Res 188:152–161, 2014). However, most recent developments of IONM technology including continuous vagal IONM and concept of staged thyroidectomy in case of loss of signal on the first side in order to prevent bilateral RLN injury may provide additional benefits which were out of the scope of this study and need to be assessed in further prospective multicenter trials.

**Electronic supplementary material:**

The online version of this article (doi:10.1007/s00423-017-1580-y) contains supplementary material, which is available to authorized users.

## Introduction

Intermittent intraoperative nerve monitoring (I-IONM) made its debut in thyroid surgery in the late 1960s with promise to reduce procedure iatrogenic nerve injury [1]. I-IONM has gained popularity in recent years with ever increasing pressures on surgeons for complication-free procedures. Despite its increasing use, I-IONM is still presently considered an adjunctive tool during thyroid surgery, taking a secondary role behind the gold standard of direct recurrent laryngeal nerve (RLN) visualization [2]. Preoperative and postoperative laryngoscopic assessment of vocal cord function should also be appraised to determine baseline and postoperative function. This supplementary role of I-IONM is supported by the recommendations set forth by the German Association of Endocrine Surgeons’ guidelines for thyroid disease and supported by the International Intraoperative Monitoring Study Group’s international standards guideline statement [2, 3]. It has also been proposed that I-IONM could play a more integral role in thyroid surgery during primary operations of high-risk patients, e.g., for retrosternal goiter, toxic goiter, Hashimoto’s thyroiditis, and Graves’ disease, or in patients undergoing revision surgery for recurrent goiter or local recurrence of thyroid cancer [4–6]. Various anatomic anomalies of the RLN such as extralaryngeal branching also pose a unique threat to the use of direct visualization and can potentially be more effectively identified with I-IONM [4]. Although many attempts have been made in recent years to statistically demonstrate the reliability of I-IONM as an essential tool for RLN identification, individual clinical studies have produced conflicting results and meta-analyses have yet to establish a uniformly acceptable conclusion. Several meta-analyses conducted in the last 5 years corroborate the current notion that I-IONM should not be incorporated into the standard of care for thyroid surgery [7–11]. Three analyses conducted by Zheng et al., Yang et al., and Wong et al. demonstrated just the opposite, in that there were significant benefits of I-IONM use [6, 12, 13]. Individual studies such as Thomusch et al. [14] and Barczynski et al. [15] have also contributed to these conflicting results.

It has been noted that from a financial perspective, I-IONM does not become justifiably cost effective unless it is able to achieve a 50.4% reduction in injuries compared to traditional direct visualization [16, 17]. Additionally, it does not significantly reduce operative time [16, 17]. Results of statistically significant injury reduction have been largely mixed and inconsistent with no obvious trend supporting movement towards full-time I-IONM use [13] or I-IONM as purely adjunctive worth [7–10]. Determining whether an improvement in transient vocal fold palsy (VFP) alone or a reduction in permanent VFP is required for implementing widespread I-IONM use is another point for debate.

A study by Sturgeon et al. indicated that approximately 37% of surgeons either routinely or in select cases use I-IONM during thyroid procedures [18]. I-IONM use according to Sanabria et al. and Barczynski et al. is also stratified based on equipment availability, experience, and surgeon age [11, 19]. Injury to the RLN during thyroid operations is notably one of the most severe postoperative complications patients experience [4, 9]. VFP is also the most frequent citing cause for litigation post thyroidectomy, as well as a significant detriment to patient quality of life [20]. Injuries bear a vast range of severity from unilateral transient VFP causing hoarseness to permanent bilateral VFP resulting in airway obstruction requiring tracheostomy [10]. Rates of transient VFP and permanent VFP have been reported as 9.8 and 2.3%, respectively [21].

Technological advances in I-IONM are occurring and the time is likely approaching when I-IONM will become standard practice for thyroid surgery patients. Yarborough et al. cite that I-IONM can play a vital role in three ways for surgeons: substantiating decisions in cases of aberrant anatomy and pathology, routine intraoperative RLN identification, and assessing postoperative RLN function [22]. Preserving RLN function is a top priority, and affording surgeons the proper information and opportunity to use all available technology to lower the injury rate is paramount. The aim of our review is to directly compare the previously conducted meta-analyses on the use of I-IONM versus direct RLN visualization by assessing rates of VFP. The ultimate goal of this analysis is to provide clarification of the differing conclusions about I-IONM use that have been presented in literature to date and put them in a perspective of rapidly developing innovations like continuous IONM technology or concept of staged thyroidectomy previously not evaluated in any of the meta-analyses. We hope that this review will serve to better guide the standards of clinical therapy and the use of I-IONM in future operative procedures.

## Methods

### Search strategy

Through February 2017, a database search was performed through PubMed, ScienceDirect, EMBASE, BIOSIS, SciELO, Web of Science, and Cochrane Library in order to identify eligible articles for the review. The search strategy employed for PubMed is presented in Table 1. No date limits or language restrictions were applied. The references in the included articles were also extensively searched. The Preferred Reporting Items for Systematic Reviews and Meta-Analyses (PRISMA) guidelines were strictly followed throughout this systematic review (Supplemental Item 1) [23].

**Table 1:** Search strategy for PubMed

No. 1	(Recurrent Laryngeal Nerve[Title/Abstract]) OR nervus laryngeus recurrens[Title/Abstract]
No. 2	(((((((Neuromonitoring[Title/Abstract]) OR Nerve monitoring[Title/Abstract]) OR Neural monitoring[Title/Abstract]) OR Real-time monitoring[Title/Abstract]) OR Electrophysiologic monitoring’[Title/Abstract]) OR Monitoring[Title/Abstract]) OR electromyography[Title/Abstract]) OR IONM[Title/Abstract]
No. 3	#1 AND #2
No. 4	((Systematic Review[Title/Abstract]) OR Meta-Analysis[Title/Abstract]) OR Review[Title/Abstract]
No. 5	#3 AND #4

### Study selection criteria

Studies were deemed eligible for inclusion if they were a meta-analysis of randomized control trials or observational studies (prospective or retrospective) comparing I-IONM to direct visualization of the RLNs during thyroidectomy with data reporting incidence of any type of VFP. The exclusion criteria included (1) systematic reviews not conducting meta-analysis or pooling of the data; (2) meta-analysis reporting incomplete data; and (3) conference abstracts, narrative reviews, commentary, or non-peer reviewed publications.

### Eligibility assessment

All studies were independently assessed for eligibility by two reviewers (B.M.H & J.V.). Any disparities arising during the assessment were resolved by a consensus among all the reviewers, after consulting with the authors of the original study, if possible. All full-text articles published in languages not spoken fluently by the authors were translated for further eligibility assessment by medical professionals fluent in both English and the original language of the manuscript.

### Data extraction

Data were independently extracted from the included analyses by three independent reviewers (J.V., P.P., B.S.). Basic data included demographic information such as year, country, studies included in their analysis, databases searched, and study design. The primary outcomes examined in this systematic review were the incidence of transient, persistent, and overall RLN injury.

### Quality assessment

Quality assessment of the included studies was conducted by two independent reviewers (J.V., B.M.H.). Any disagreements were resolved by discussion until mutual consensus or involving a third reviewer (M.J.G.). The Assessment of Multiple Systematic Reviews (AMSTAR) method was used for study appraisal [24]. The AMSTAR performed included 11 criteria and has become the standard for meta-analysis and systematic review quality assessment [25].

### Selection of best evidence

Evaluation of included meta-analyses was performed through the implementation of a Jadad decision algorithm [26]. Utilization of the Jadad algorithm allowed for the discernment between varying methodological practices in different meta-analyses and systematic reviews ranging from data-extraction, inclusion/exclusion criteria, to statistical analyses performed [26]. The Jadad system is designed to allow for a clear assessment of the evidence presented in a given analysis and allows for the determination of which study provides the best overall evidence given the present information.

## Results

### Study identification and characteristics of included studies

**Fig. 1:**
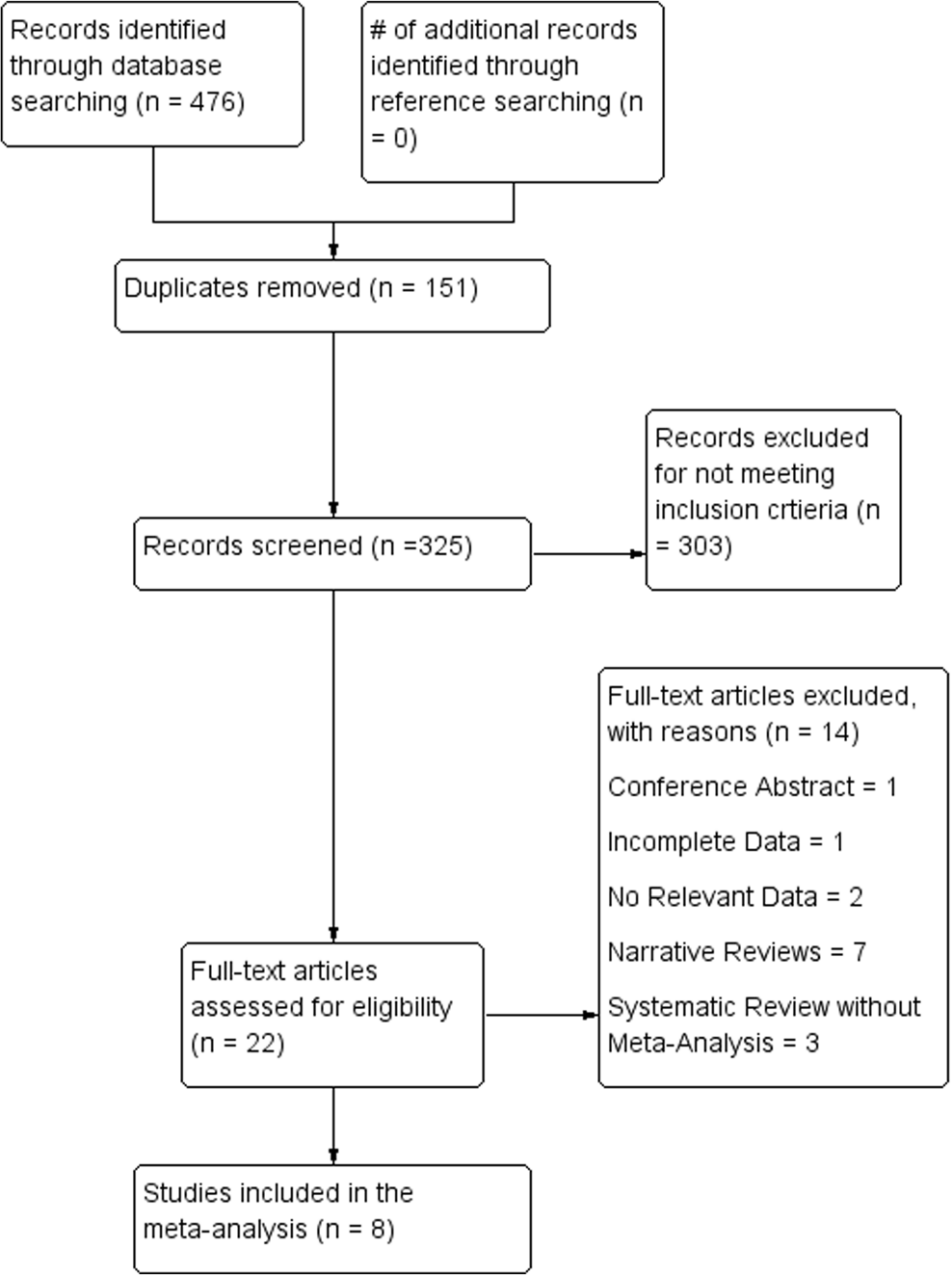
PRISMA flowchart of study identification and inclusion in the meta-analysis

A summary of the flow of studies through the systematic review is presented in Fig. 1. A search through the major electronic databases identified 476 articles; none were identified when the references of the included studies were searched. After 151 duplicates had been excluded and 325 records screened, 22 articles were further assessed for eligibility by full text. Among these, 14 were excluded and 8 were included in the review. One meta-analysis by Rulli et al. [27] was excluded due to incomplete data with respect to details of their samples.

The characteristics of the meta-analyses included in this study are detailed in Table 2. Included studies ranged in time from 2011 to 2017 and included between 6 and 23 studies in their analyses. Table 3 provides cross-linking of the original studies included in the 8 meta-analyses reviewed in this present work. The original studies included in the different meta-analyses varied significantly despite the research largely targeting the same primary outcome. Additionally, the meta-analysis conducted by Wong et al. [6] focused entirely on procedures which were deemed “high-risk,” such as those undergoing repeat operation and operations due to significant pathology.

**Table 2:** Characteristics of included studies

Study ID	Journal	Literature search valid through	Published	Number of included studies					Case series
				Total	RCT	Comparative			
						Total	Prospective	Retrospective	
Higgins 2011	The Laryngoscope	July 2008	2011	9 (+34)	1	8	4	4	34
Lombardi 2016	Surgery	August 2014	June 2016	14	4	10	5	5	0
Malik 2016	World J Surg	Unknown	June 2016	15 (+2)	1	14	Unknown	Unknown	2
Pisanu 2014	J Surgical Research	August 2013	2014	20	3	17	7	10	0
Sanabria 2013	Eur Arch Otorhinolaryngol	December 2012	May 2013	6	6	0	0	0	0
Zheng 2013	J Formos Med Assoc	April 2011	2013	14	2	12	8	4	0
Yang 2017	International J Surgery	July 2016	2017	23	4	19	6	13	0
Wong 2017^a^	International J Surgery	September 2015	2017	10	0	10	2	8	0

*RCT* randomized controlled trial

^a^Conducted solely on “high-risk” patients undergoing re-operations or significant pathology

**Table 3:** Citation matrix of primary studies included in previous meta-analyses (excluding case series)

	Higgins 2011	Lombardi 2016	Malik 2016	Pisanu 2014	Sanabria 2013	Zheng 2013	Yang 2017	Wong 2017
Agha 2008	–			–		–	–	
Alesina 2012		–		–			–	–
Atallah 2009				–				–
Barczynski 2009	–	–	–	–	–	–	–	
Barczynski 2011				–				–
Barczynski 2012		–			–			
Barczynski 2014		–					–	–
Brauckhoff 2002						–		
Calo 2013				–				
Calo 2014		–						–
Calo 2016							–	
Chan 2006	–	–	–	–		–	–	–
Chiang 2008			–				–	
Danschutter 2015							–	
De Falco 2014		–						
Diongi 2009		–		–	–		–	
Dralle 2004	–	–	–	–		–		–
Duclos 2011			–	–			–	
Frattini 2010			–	–		–	–	–
Gremillion 2012				–			–	
Hei 2016							–	
Khaled 2012					–			
Lifante 2009					–			
Netto 2007	–		–	–		–	–	
Page 2015							–	
Prokopakis 2013								–
Robertson 2004	–		–	–		–	–	
San 2010						–		
Sari 2010		–		–	–	–	–	
Shindo 2007	–			–			–	
Snyder 2013			–					
Stevens 2012		–		–			–	
Terris 2007	–	–	–	–		–	–	
Thomusch 2002		–	–			–		
Witt 2005	–	–		–		–	–	
Xie 2016							–	
Yarbrough 2004			–	–		–	–	–

### Search methodology

Of the eight studies included, they differed significantly in their literature search methodology and the databases in which they included. The databases utilized by each included meta-analysis are detailed in Table 4. All studies included a literature search of MEDLINE; however, all other databases were not universally searched among the included studies.

**Table 4:** Databases searched in each original meta-analysis

Databases	Higgins 2011	Lombardi 2016	Malik 2016	Pisanu 2014	Sanabria 2013	Zheng 2013	Yang 2017	Wong 2017
MEDLINE	–	–	–	–	–	–	–	–
EMBASE	–		–	–	–	–	–	–
Cochrane	–	–		–	–	–	–	–
clinicaltrials.gov	–							
The National Guideline Clearinghouse	–							
Scopus		–						
Google Scholar				–				
Ovid								
LILACS					–			

### AMSTAR quality assessment

The results of the AMSTAR checklist are presented in detail in Table 5. Studies uniformly developed study designs prior to execution as well as performed systematic literature searches of their intended databases. Quality assessment was largely overlooked in many cases and rarely did quality factor into decision-making when it came to conclusions.

**Table 5:** AMSTAR Criteria for included meta-analyses

Items	Higgins 2011	Lombardi 2016	Malik 2016	Pisanu 2014	Sanabria 2013	Zheng 2013	Yang 2017	Wong 2017
Was an “a priori” design provided?	1	1	1	1	1	1	1	1
Was there duplicate selection and data extraction?	1	1	0	1	1	1	1	1
Was a comprehensive literature search performed?	1	1	1	1	1	1	1	1
Was the status of publication used as an inclusion criterion?	0	0	0	0	0	1	0	0
Was a list of included/excluded studies provided?	0	0	0	0	0	0	0	0
Were the profiles of the included studies provided?	1	1	0	1	1	1	1	1
Was the methodological quality of the included studies evaluated and documented?	0	1	0	0	1	1	0	1
Was the specific quality of the included studies used appropriately in formulating conclusions?	0	0	0	0	1	0	0	0
Were the methods used to combine the findings of studies appropriate?	1	1	0	1	1	1	1	1
Was the publication bias evaluated?	1	0	0	0	1	1	1	1
Were the conflicts of interest stated?	1	0	0	1	0	0	1	1
Total score	**7**	6	2	6	8	8	7	8

### Overview of primary and secondary outcomes

The results of the three primary outcomes, overall, transient, and persistent RLN injury, are summarized in Tables 6, 7, and 8, respectively.

**Table 6:** Overall incidence of vocal fold palsy between IONM and direct visualization

	Overall VFP (overall incidence)							
	IONM			Visual identification only			Odds ratios	
	*n* = (nerves)	VFP	%	*n* = (nerves)	VFP	%	OR (95% CI)	*p* value
Higgins 2011 (excluding case series)	20,500	729	3.56	7939	325	4.09	0.87 (0.73–1.03)	0.11
Higgins 2011 (case series)	7435	253	3.4	29,440	845	2.87	1.32 (0.77–2.27)	0.31
Malik 2016	25,843	822	3.18	18,732	718	3.83	NR	–
Pisanu 2014	24,038	834	3.47	11,475	421	3.67	0.94 (0.83–1.06)	0.471
Sanabria 2013	1446	40	2.77	1466	70	4.77	NR	–
Zheng 2013	23,298	786	3.37	12,898	485	3.76	0.74 (0.59–0.92)	0.007
Yang 2017	8668	273	3.15	8535	373	4.37	0.81 (0.66–0.99)	0.041
Wong 2017 (high-risk thyroidectomy)	6155	151	2.5	4460	201	4.5	1.42 (1.12–1.79)	0.003
Wong 2017 (re-operation thyroidectomy)	1751	78	4.45	1497	114	7.61	1.48 (1.06–2.06)	0.021
Wong 2017 (thyroidectomy for malignancy)	2468	52	2.11	1596	55	3.45	1.52 (1.00–2.31)	0.05

*IONM* intraoperative nerve monitoring, *VFP* vocal fold palsy, *OR* odds ratio, *NR* not reported

**Table 7:** Incidence of transient vocal fold palsy between IONM and direct visualization

	Transient VFP							
	IONM			Visual identification only			Odds ratios	
	*n* = (nerves)	Trans. VFP	%	*n* = (nerves)	Trans. VFP	%	OR (95% CI)	*p* value
Higgins 2011 (excluding case series)	20,500	552	2.69	7939	234	2.95	0.94 (0.80–1.10)	.44
Higgins 2011(case series)	7435	213	2.86	29,440	697	2.37	1.43 (0.86–2.38)	.16
Pisanu 2014	24,038	630	2.62	11,475	312	2.72	0.95 (0.82–1.10)	0.552
Sanabria 2013	1446	32	2.21	1466	58	3.96	NR	–
Zheng 2013	23,298	596	2.56	12,898	350	2.71	0.80 (0.65–0.99)	.04
Yang 2017	8668	158	1.82	8535	220	2.58	0.76 (0.61–0.94)	0.013
Wong 2017 (high-risk thyroidectomy)	3017	71	2.5	3332	129	3.9	1.47 (1.07–2.00)	0.016
Wong 2017 (re-operation thyroidectomy)	812	35	4.31	1188	75	6.31	1.49 (0.95–2.33)	0.082
Wong 2017 (thyroidectomy for malignancy)	1282	21	1.64	1160	36	3.1	1.90 (1.08–3.35)	0.026

*IONM* intraoperative nerve monitoring, *VFP* vocal fold palsy, *OR* odds ratio, *NR* not reported

**Table 8:** Incidence of persistent vocal fold palsy between IONM and direct visualization

	Persistent VFP							
	IONM			Visual identification only			Odds ratios	
	*n* = (nerves)	Pers. VFP	%	*n* = (nerves)	Pers. VFP	%	OR (95% CI)	*p* value
Higgins 2011 (excluding case series)	20,500	167	0.81	7939	79	0.99	0.88 (0.66–1.16)	.36
Higgins 2011(case series)	7435	42	0.56	29,440	146	0.5	0.95 (0.43–2.10)	.9
Lombardi 2016 (NRS; 6 months)	21,197	158	0.75	11,093	94	0.85	NR	–
Lombardi 2016 (NRS; 12 months)	3152	21	0.67	3378	35	1.03	NR	–
Lombardi 2016 (NRS; overall)	24,349	179	0.73	14,471	129	0.89	NR	–
Lombardi 2016 (RCTs)	1465	8	0.55	1458	12	0.82	NR	–
Pisanu 2014	24,038	190	0.79	11,475	106	0.92	0.88 (0.69–1.14)	1
Sanabria 2013	1446	8	0.55	1466	12	0.82	NR	–
Zheng 2013	23,508	183	0.78	13,097	126	0.96	0.80 (0.62–1.03)	.09
Yang 2017	8668	58	0.67	8535	91	1.06	0.78 (0.55–1.09)	0.146
Wong 2017 (high-risk thyroidectomy)	6095	80	1.31	4399	72	1.64	1.33 (0.94–1.88)	0.104
Wong 2017 (re-operation thyroidectomy)	1691	43	2.54	1436	39	2.72	1.40 (0.87–2.27)	0.171
Wong 2017 (thyroidectomy for malignancy)	2468	31	1.26	1596	19	1.19	1.13 (0.61–1.11)	0.696

*IONM* intraoperative nerve monitoring, *VFP* vocal fold palsy, *OR* odds ratio, *RCT* randomized controlled trial, *NRS* non-randomized studies, *NR* not reported

### Heterogeneity assessment

Heterogeneity of the primary outcomes studied in this analysis was extracted and recorded. A detailed breakdown of heterogeneity data is available in Table 9. The *I* ^2^ statistic was utilized in 6/8 [9–11, 13, 12, 6] included studies and was calculable in a fifth [8]. No heterogeneity was recorded by Malik et al. [7].

**Table 9:** Heterogeneity (*I* ^2^ statistic) of primary outcomes included in the meta-analysis

	Higgins 2011	Lombardi 2016	Malik 2016	Pisanu 2014	Sanabria 2013	Zheng 2013	Yang 2017	Wong 2017
Overall VFP	39%	0%	–	0%	–	33%	21%	6.3%
Transient VFP	24%	0%	–	0%	31%	15%	0%	6.3%
Persistent VFP	0%	0%	–	0%	0%	0%	0%	0%

*–* no heterogeneity data reported or calculable, *VFP* vocal fold palsy

### Results of Jadad decision algorithm

**Fig. 2:**
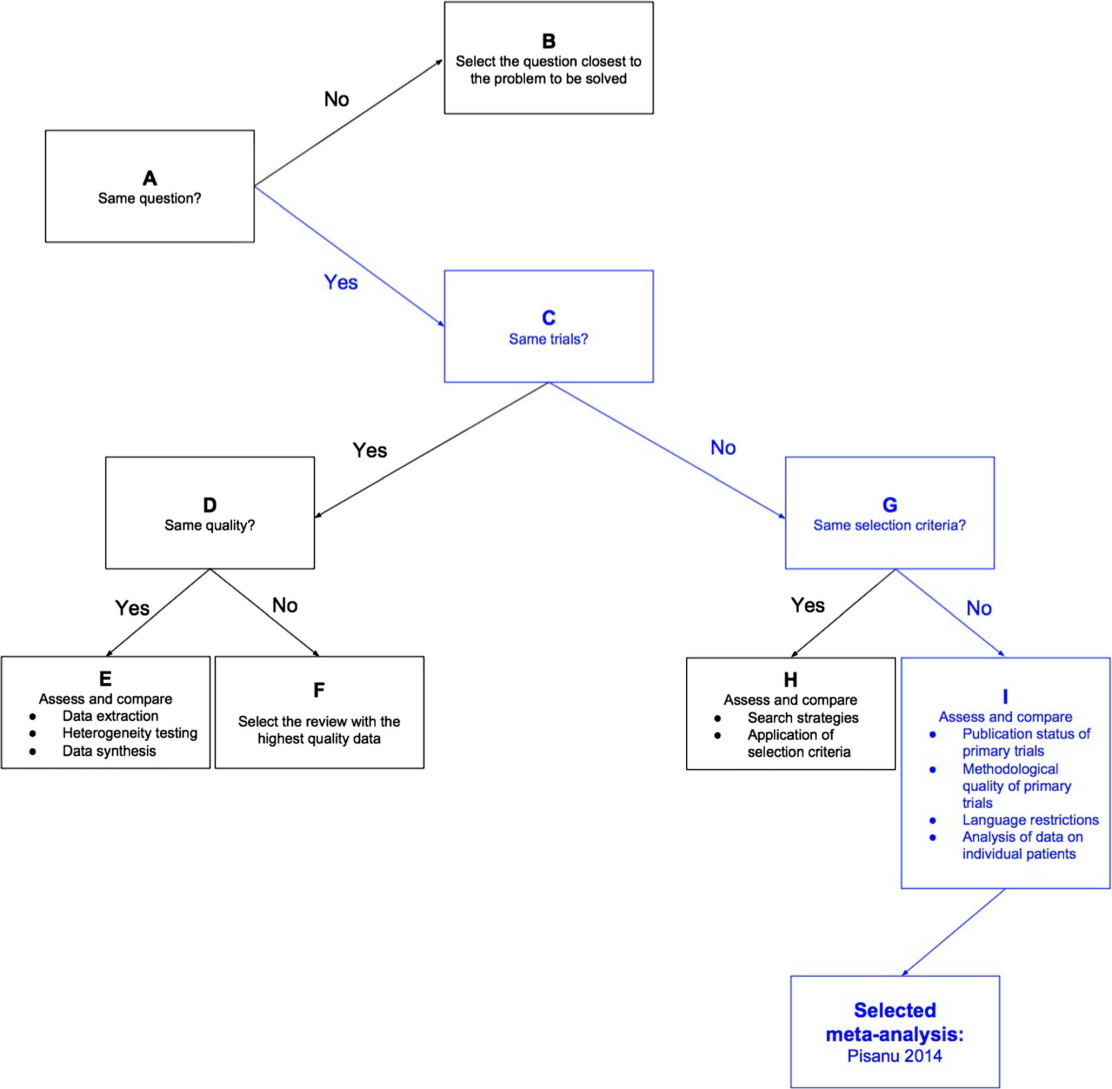
Jadad decision algorithm

Included meta-analyses were analyzed for methodological quality using a Jadad algorithm (Fig. 2). The Jadad assessed quality based on their utilization of the same primary outcome, studies included, and selection criteria. Furthermore, studies were compared on their use of included study quality in drawing conclusions, language restrictions present, and data analysis procedures. As a result, the meta-analysis with the highest quality was selected. Pisanu et al. [8] was selected and supports the notion that there is no statistically significant reduction in RLN injury between procedures with I-IONM over direct RLN visualization.

## Discussion

This comprehensive review is aimed at comparing and summarizing meta-analyses on the rates of RLN injury between groups undergoing either direct visualization or I-IONM during thyroidectomy. The meta-analysis conducted by Pisanu et al. was determined to be of the highest methodological quality when compared to the other included studies [8]. The evidence to date supports the notion that I-IONM does not provide for significant reduction in postoperative RLN injury challenging rationale for its widespread implementation [8].

Meta-analyses have long been utilized to increase the statistical power through the combination of smaller studies in hopes of uncovering meaningful data for use in clinical practice. In the case of I-IONM, the recommendations have varied widely between individual studies and in the several analyses conducted in recent years. With the continually variable conclusions being published on the subject, it becomes very difficult for clinicians and scientists alike to form meaningful practice changing policy. It is the intent of this analysis to provide a pathway for these individuals and agencies to easily navigate these conflicting studies.

In 1997, Jadad et al. devised a system with which to sift through these analyses that have come to differing conclusions [26]. It was proposed that there are several sources that could lead to these discordant results such as inclusion criteria, extraction techniques, statistical analysis procedures, and quality assessment [26].

The meta-analysis conducted by Pisanu et al. was the study selected that best reflects the present literature [8]. It can be noted in Table 3 that Pisanu et al. included the second highest number of studies in their analysis [8]. The chosen study achieved a score of 6 in the AMSTAR checklist utilized. The score was largely lowered by the lack of quality assessment of the studies that were included. There were five studies which resulted in an AMSTAR of higher value: Higgins et al. [10], Sanabria et al. [11], Zheng et al. [13], Yang et al. [12], and Wong et al. [6]. Through the utilization of the Jadad method, and assessing the number of studies included, the databases searched, journal of publication, and methodology, the authors still deemed Pisanu et al. to be the meta-analysis of highest quality [8]. The conclusion that I-IONM should be used as a purely adjunctive measure that resulted in Pisanu et al. (*p* = 0.471) [8] was largely supported by the other analyses included in this present review [Malik 2016 (*p* > 0.05) [7], Lombardi 2016 (*p* > 0.05) [9], Higgins 2011 (*p* = 0.11) [10], Sanabria 2013 (*p* = 0.15) [11]], clashing with the results of three reviews (Zheng 2013, *p* = 0.007, Yang 2017, *p* = 0.041, Wong 2017, *p* = 0.003, 0.021, and 0.05) [6, 12, 13]. The meta-analysis conducted by Wong et al. [6] demonstrated significant rates of injury reduction in both overall and transient VFP; it should however be noted that this study focused entirely on high-risk procedures of patients undergoing re-operation or those with significant pathology such as malignancy or retrosternal goiter.

I-IONM in theory appears as an ideal tool that could provide better outcomes for patients. It has been noted that with traditional intermittent (non-continuous) IONM techniques, injuries are often discovered by the machine but only after they have already occurred [28]. Until injuries can be prevented as opposed to being simply identified, this approach does not seem to be justifiable. However, results reported by Bergenfelz et al. in a database from a Swedish multicenter audit comprising 3660 patients undergoing thyroid surgery showed that RLN injury was recognized intraoperatively in only 16 (11.3%) out of 142 patients with a damaged nerve [29]. This in agreement with recent studies showing that RLN injury most often occurs to a visually observed nerve. Hence, I-IONM has a potential to improve the intraoperative RLN management by elucidating mechanisms of nerve injury. In addition, I-IONM is an effective tool in staging planned bilateral thyroid surgeries in cases of intraoperative RLN injury on the side of initial dissection [30]. This issue is of great importance in prevention of bilateral RLN injury which occurs in approximately 0.2% of patients undergoing thyroidectomy leading to significant deterioration of quality of life and medicolegal claims [31]. Bergenfelz et al. analyzed the risk of RLN palsy in a cohort of 5252 patients undergoing thyroidectomy with and without intraoperative nerve monitoring who were registered in the Scandinavian Quality Register for Thyroid, Parathyroid and Adrenal Surgery in 2009–2013 [32]. I-IONM was used in 3277 operations (62.4%), and postoperative laryngoscopy was performed in 1757 patients (33.5%) [32]. Early VFP occurred in 217 patients (4.1%), of which 3 were bilateral, all in the group without I-IONM. Permanent VFP occurred in 62 patients (1.2%). In the multivariable analysis of 1757 patients who had postoperative laryngoscopy, the use of I-IONM was not associated with a decreased risk of early VFP [OR 0.67 (95% CI 0.44–1.01)], but decreased the risk of permanent VFP [OR 0.43 (95% CI 0.19–0.93)]. Thus, data arising from a prospective register reflect to a greater extent some details of current surgical practice landscape in thyroid surgery rather than data from meta-analysis based on mixed and often inclusive of poorly reported retrospective case series.

It was noted by Chan et al. that among a survey of members of the American Association of Endocrine Surgeons, protection from litigation was cited as a primary reason for I-IONM use [33]. The addition of costly procedures to standard operative technique can only be rationalized by an improvement in clinical outcomes and should not be on the basis of legal protection [16]. The use of I-IONM is also associated with a learning curve and is predominantly utilized by surgeons under the age of 40 [11, 19]. This learning curve can be associated with a period of higher incidence of injury which should be factored into research conducted in the future [7, 34]. Hence, training and exposure to the standardized utilization of I-IONM technique during thyroid surgery by attending to one of the hands-on courses organized worldwide and accredited by the International Neural Monitoring Study Group in Thyroid and Parathyroid Surgery should be warranted to optimize the clinical benefit. Finally, the most recent developments of IONM technology including continuous vagal IONM with intraoperative real-time electromyography of the vocalis muscles and its potential to recognize the imminent RLN injury which could be prevented by modifying surgical maneuvers seem to be a very promising tool considered to be a quantum leap forward in the prevention of the RLN injury during thyroid surgery [35]. It should be underlined that any of the published meta-analyses included continuous IONM studies as they were undertaken and published later on.

This systematic review was limited by a number of factors. Broadly speaking, the progress of IONM technology and the methods utilized varied in the included meta-analyses. It is likely that as IONM technology improves over time, there will be increasing utility for not only identifying the RLN but also preventing the RLN injury. Older studies may introduce a level of bias into meta-analyses because of the falsely depressed levels of RLN identification and non-standardized use of the I-IONM technique both for nerve identification and for prognostication of neural function. In addition, postoperative laryngoscopy was used on a select basis in many centers leading to underestimation of the true prevalence of the RLN injury. It is important in future investigations to reduce this potential bias through subgroup analysis on the basis of study year, use of the standardized approach to IONM, and postoperative laryngoscopy, as well as potentially the equipment used. With regard to the varying methodology, studies varied greatly on their search strategies, many of which did not include foreign language articles. Future meta-analysis on this topic should use a more rigorous methodology. Numerous issues with the originally included primary studies such as bias, study design, and sample heterogeneity may have had significant impacts on the final interpretations of their results.

There is a need for continuing investigation into the use of IONM as a primary method for RLN identification and prevention of postoperative complications. IONM is a developing technology, and likely with improvements, there will come a time where it does provide the necessary reduction in injuries to warrant its widespread implementation [7]. Additionally, original studies and new meta-analyses are needed to further investigate the use of IONM specifically in cases of reoperation as has been initiated by Wong et al. [6]. Many studies have shown insignificant improvement in postoperative complications in primary procedures, while demonstrating potential use in secondary operations [7, 33, 36].

## Conclusion

To date, I-IONM has not achieved a significant level of RLN injury reduction as shown by the meta-analysis conducted by Pisanu et al. [8]. However, most recent developments of IONM technology including continuous vagal IONM and concept of staged thyroidectomy in case of loss of signal on the first side in order to prevent bilateral RLN injury may provide additional benefits which were out of the scope of this study and need to be assessed in further prospective multicenter trials.

## Electronic supplementary material

Supplement Item 1 PRISMA 2009 Checklist (PDF 65 kb)
